# Application of Probabilistic Genotyping Software to Paternity Cases Involving Low-Template DNA †

**DOI:** 10.3390/genes17020187

**Published:** 2026-02-01

**Authors:** Alessia Riem, Elena Chierto, Federica Bertolotto, Marco Parnigoni, Serena Aneli, Carlo Robino

**Affiliations:** Department of Public Health Sciences and Pediatrics, University of Turin, 10126 Turin, Italy; alessia.riem@unito.it (A.R.); elena.chierto@unito.it (E.C.); bertolottof@gmail.com (F.B.); marco.parnigoni@unito.it (M.P.); serena.aneli@unito.it (S.A.)

**Keywords:** kinship analysis, paternity testing, low-template DNA, probabilistic genotyping

## Abstract

**Background:** Interpreting short tandem repeat (STR) profiles from low-template DNA (LT-DNA) requires consideration of the stochastic phenomena that can affect the reliability of genotypes. Although several probabilistic genotyping tools have been developed to model such uncertainties, most have only been used for direct comparisons between persons of interest and crime scene samples. Their application to kinship testing involving LT-DNA has received comparatively little attention. **Methods**: We evaluated the performance of two PGS, EuroForMix (EFM) and EFMrep, which support alternative hypotheses with relatedness, by comparing them with a standard paternity testing software (Familias) in 33 paternity cases involving LT-DNA samples categorised as ‘mildly’ (MD) or ‘highly’ (HD) degraded based on the quality of the STR profiles. The samples included formalin-fixed paraffin-embedded tissues, bone specimens, and stains collected from personal items. Pedigrees with (‘trio’) and without (‘duo’) maternal information were considered. **Results**: In MD and HD duos, the likelihood ratios (LRs) obtained with EFMrep were significantly higher compared to other software. In trios, Familias produced significantly higher LRs than PGS for MD samples, whereas the three software performed comparably for HD samples. Notably, in HD trios, EFMrep was the software most likely to maximise LR values, which were above 10,000 in 60% of the cases, compared to 50% of EFM and 40% of Familias. **Conclusions**: These findings provide preliminary evidence of the potential and limitations of using PGS for kinship assessments involving LT-DNA specimens.

## 1. Introduction

In forensic genetics, the PCR amplification of short tandem repeat (STR) loci in low-template DNA (LT-DNA) samples is affected by stochastic variation [1]. This can lead to the unbalanced amplification of alleles, generating so-called drop-out and drop-in events. Drop-out events consist of the failure to amplify an allele that is actually present in the genotype of the source individual, while drop-in events consist of the observation of additional alleles that are not part of the source individual’s genotype [2]. The occurrence of stochastic events is favoured by suboptimal amounts of input DNA in the amplification reactions of STR loci and by the reduced number of DNA templates accessible to PCR primers in cases of DNA degradation [3]. Another feature of LT-DNA samples that may complicate interpretation is the possible occurrence of large stutters [4]. These are artefactual peaks, typically one repeat unit shorter or longer than the actual allelic peak, that are the result of polymerase slippage during the process of amplifying repetitive STR sequences [5]. These peaks may be mistaken for real alleles in unbalanced PCR results.

Previous studies have shown that all these PCR artefacts can be interpreted within the framework of likelihood ratio (LR) calculations [6]. Probabilistic genotyping software (PGS) that can accommodate drop-out, drop-in and stutter events has been developed and validated accordingly [7,8]. This software can also handle the other complexities of forensic STR profiles, such as mixtures involving multiple contributors. In their most advanced form, known as ‘continuous’ methods, PGS can use quantitative information derived from peak heights in the DNA profile to calculate the probability of the observed peaks, given all possible genotype combinations for the individual contributors [9]. However, most of this software has been specifically designed to facilitate the interpretation of direct matches between a reference profile and a sample of interest in criminal investigations. Less attention has been given to probabilistic genotyping in the context of kinship and paternity testing [10], even though LT-DNA samples are not uncommon in such cases. Consider, for instance, cases of alleged paternity involving a deceased father, where the STR profile must be derived from archival material consisting of formalin-fixed, paraffin-embedded (FFPE) tissue, whose DNA is often fragmented due to the degradative effect of formalin [11]. Similarly, DNA degradation due to biochemical post-mortem modifications of the body is expected in exhumed human remains used for paternity testing [12]. Post-mortem and peri-mortem events, such as those involving fire [13], can also lead to DNA degradation in human remains. This has an impact on kinship calculations, which are carried out when a reference sample of the missing person is unavailable and DNA comparisons with relatives are needed for identification purposes [14].

Software capable of calculating LR in paternity and kinship testing, incorporating features such as the ability to consider maternal genotype information, mutation rates, and different mutation models for STRs, has existed for a considerable time [15]. More recently, these software programs have progressively been implemented to include the probability of drop-out events, which can result in apparent mismatches between genotypes [16,17]. However, software that can fully handle quantitative information to estimate genotype probabilities given all possible genotype sets and combine replicate analyses of STR loci into a single likelihood ratio (LR) value is still limited in availability and diffusion [18,19].

The current recommendations for the biostatistical evaluation of paternity, as developed by the International Society for Forensic Genetics (ISFG), do not address LT-DNA cases and the associated stochastic events [20]. In our laboratory, at least two STR amplification replicates are carried out for paternity/kinship testing of potentially LT-DNA samples, i.e., those derived from unconventional sources other than reference buccal swabs and fresh blood/tissue samples. A ‘consensus’ profile [1] is then obtained and analysed using the drop-out function available in the Familias software [17].

Some continuous PGS support the option of considering the hypothesis that a relative of the person of interest (POI) from whom the reference profile was obtained is the actual donor of the crime stain, rather than the POI him/herself [21]. It is therefore possible to calculate LR values given the proposition that the LT-DNA profile of interest comes from the father–child of a reference person, whose DNA profile is available and uncontested, rather than from a random individual [22]. With this in mind, we decided to investigate the possibility of applying such software to paternity cases involving LT-DNA samples. The software programs considered here were EuroForMix (EFM) [23] and EFMrep [24]. The latter is an extension of EuroForMix, which enables DNA profiling results obtained with different STR kits to be combined. Like EFM, it includes the option of specifying related individuals in alternative propositions.

## 2. Materials and Methods

### 2.1. Samples

A total of 33 LT-DNA samples from 15 different cases submitted to the laboratory between 2019 and 2025 were analysed. Research was authorised by the Bioethics Committee of the University of Turin (Authorisation No. 0532888, 18 July 2025). The cases included: (i) paternity tests involving a deceased alleged father for whom ante-mortem samples were available and consisted of FFPE samples (*n* = 12 samples from 7 cases) or dry blood/saliva stains (*n* = 6 samples from 2 cases); (ii) identification of unknown skeletal remains through comparison with reference samples belonging to an alleged first-degree relative (*n* = 15 samples from 6 cases). Maternal information was available for 21 samples (‘trio’ cases) and missing in 12 samples (‘duo’ cases).

### 2.2. DNA Extraction

DNA extraction was performed using different methods depending on the starting material: (i) FFPE samples: Two to three sections of tissue, 5–10 μm thick, were cut via a microtome from each FFPE sample and deparaffinized using Deparaffinization Solution (Qiagen, Hilden, Germany) followed by DNA purification with a QIAamp DNA FFPE Tissue kit (Qiagen, Hilden, Germany) performed either manually (2019–2021) or automatically (2022–2025) with QIACube Connect (Qiagen, Hilden, Germany). (ii) Bone: The PrepFiler BTA Forensic Kit (ThermoFisher Scientific, Waltham, MA, USA) was used, following the manufacturer’s instructions. The procedure started from 50 mg of bone powder previously obtained through mechanical scraping followed by pulverisation in liquid nitrogen. (iii) Dried stains: The QIAamp DNA Investigator Kit (Qiagen, Hilden, Germany) was used, following the manufacturer’s instructions, either manually (2019–2021) or automatically (2022–2025) with QIACube Connect (Qiagen, Hilden, Germany). (iv) Reference samples: Buccal swabs were extracted using the Investigator STR GO! lysis buffer (Qiagen, Hilden, Germany) following the manufacturer’s instructions.

### 2.3. DNA Quantification

Total human DNA and male-specific DNA isolated from the LT-DNA samples were quantified by qPCR using the Plexor^®^ HY System Kit (Promega, Madison, WI, USA) and the CFX96 Touch^TM^ Real-Time PCR Detection System (Bio-Rad Laboratories, Hercules, CA, USA). Results were analysed with the Plexor Forensics software version 1.6.0 (Promega, Madison, WI, USA).

### 2.4. Genotyping

Samples were amplified with the PowerPlex ESI 17 Fast (Promega, Madison, WI, USA) (‘ESI’) and PowerPlex ESX 17 Fast (Promega, Madison, WI, USA) (‘ESX’) kits, targeting the same panel of STR loci with alternative PCR primers. Primers are designed so that STRs with shorter amplicon sizes in one kit have larger amplicon sizes in the other, and vice versa, so that the combination of the two kits maximises the number of genotyped STR loci [25]. For LT-DNA samples, duplicate amplifications were performed using either the ESI or ESX kit, in addition to a single amplification carried out with the kit not used for the duplicate reactions. The optimal amount of input DNA according to the manufacturer’s instructions (0.5 ng) was employed. When this was not possible, due to reduced DNA concentration in LT-DNA samples, the maximum reaction volume available in PCR for input DNA was used. STR genotyping was performed by capillary electrophoresis using the SeqStudio Genetic Analyzer for HID (ThermoFisher Scientific, Waltham, MA, USA). Data analysis was carried out with GeneMapper ID-X software version 1.7 (ThermoFisher Scientific, Waltham, MA, USA). The analytical threshold (AT), separating reported alleles from electrophoretic baseline noise was set at 50 relative fluorescence units (RFU) for all STR kits.

The degradation status of LT-DNA samples was assessed by considering, in each STR profile, the largest amplicon product with RFU peak height associated with an expected probability of drop-out <5% (5% stochastic threshold, 5ST) [26] according to logistic regression experiments [7]. Samples were classified as highly degraded (HD) when the 5ST largest amplicon length was <200 bp and mildly degraded (MD) when it was ≥200 bp.

### 2.5. Software Programs for LR Calculation

All LR calculations were performed using the allelic frequencies observed in the Italian population [27,28], except for locus SE33 [29], and by applying a 0.01 F_ST_ correction for population structure [30]. The interpretation of LR values was performed taking into account national guidelines for kinship testing, suggesting a threshold of 10,000 for DNA results to be acceptable in court as proof of paternity [31,32].

The specific parameters set according to the characteristics of the software Familias version 3.3.1, EuroForMix version 4.0.8, and EFMrep version 1.1.0 (Table 1) are summarised in the following sections.

#### 2.5.1. Familias

A ‘consensus’ profile was obtained using the ‘2×’ approach [33], which exclusively considers the alleles detected at least twice in the PCR experiments consisting, in this case, of two replicates performed with the same STR kit (ESI or ESX). The criteria for establishing the consensus profile and to assign the corresponding drop-out probabilities in Familias are outlined in Table 2. The probability distribution of drop-out was determined through internal validation and logistic regression (Supplementary Figure S1) [7].

Mean mutation rates for male and female individuals, as described in the 2003 report of the American Association of Blood Banks [34], were used. For loci not considered in that report, a standard mutation rate of 0.2% was adopted. The ‘equal probability’ mutation model was applied in all calculations.

In trio cases, calculations were performed both with and without considering maternal information.

#### 2.5.2. EFM

LT-DNA samples underwent two PCR replicates with the same STR kit (ESI or ESX). The two alternative hypotheses used in the calculations were: (i) Hp: sample comes from an individual unrelated to the reference person; and (ii) Hd: sample comes from the parent/child of the reference person.

Lambda parameters to model drop-in peak height were calculated with the EFM ‘Fit-in drop-in data’ tool, based on internal validation data obtained for the ESI (0.038) and ESX (0.061) STR kits. The exponential drop-in functions for the two STR kits are displayed in Supplementary Figure S2.

The reciprocal of the maximum likelihood estimation (MLE) LR value obtained for the combination of parameters (degradation, forward and backward stutter) that maximised the adjusted loglikelihoods of the competing propositions was then used for comparisons between software.

#### 2.5.3. EFMrep

Each LT-DNA sample was subjected to amplification using two PCR replicates with different STR kits (ESI and ESX). Lambda values were the same as those for EFM. The same combination of parameters maximising LR according to the results obtained with EFM using a single STR kit was selected, and it was assumed that they also applied to the STR kit, which had not been used for EFM calculations. As EFMrep, differently to EFM, supports relatedness as the numerator proposition, the two alternative hypotheses were: (i) Hp: sample comes from the parent/child of the reference person; and (ii) Hd: sample comes from an individual unrelated to the reference person.

The obtained MLE LR value was then used for comparisons between software.

### 2.6. Biostatistical Analysis

Statistical comparisons of data distributions were computed using the Wilcoxon signed-rank test. Distribution correlations were evaluated using the Pearson correlation. A statistical significance threshold of *p* < 0.05 was applied. All statistical analyses were conducted using R statistical software (version 4.4.1) [35].

## 3. Results

Of all 33 LT-DNA samples included in the study, 61% were classified as HD (n = 20) and 39% as MD (n = 13), according to the 5ST stochastic threshold derived from electropherograms. Separated analyses were conducted for duo and trio pedigrees. LT-DNA paternity cases in which maternal information was available (n = 21) were used for calculations in both the trio and the duo scenario, disregarding maternal data in the latter case. The general characteristics of LT-DNA samples and the results of the software analysis are detailed in Supplementary Table S1. The number of loci that were informative of the genotype for LR calculations using the three different software is shown in Table 3. For Familias, only loci of the consensus profile with at least one detected allele were considered, whereas for EFM and EFMrep, loci with at least one allele above AT in one of the two PCR replicates were counted. It was seen that, for HD samples, the number of informative STR loci was significantly lower in Familias compared to EFM (*p* = 0.0002) and EFMrep (*p* = 0.0002). No differences between the three software were seen for MD samples. In HD samples, a significant positive correlation was observed between the number of informative STR loci and the obtained LR values in Familias (r = 0.75, *p* = 0.0002), EFM (r = 0.76, *p* = 0.0001), and EFMrep (r = 0.48, *p* = 0.0323).

**Figure 1:**
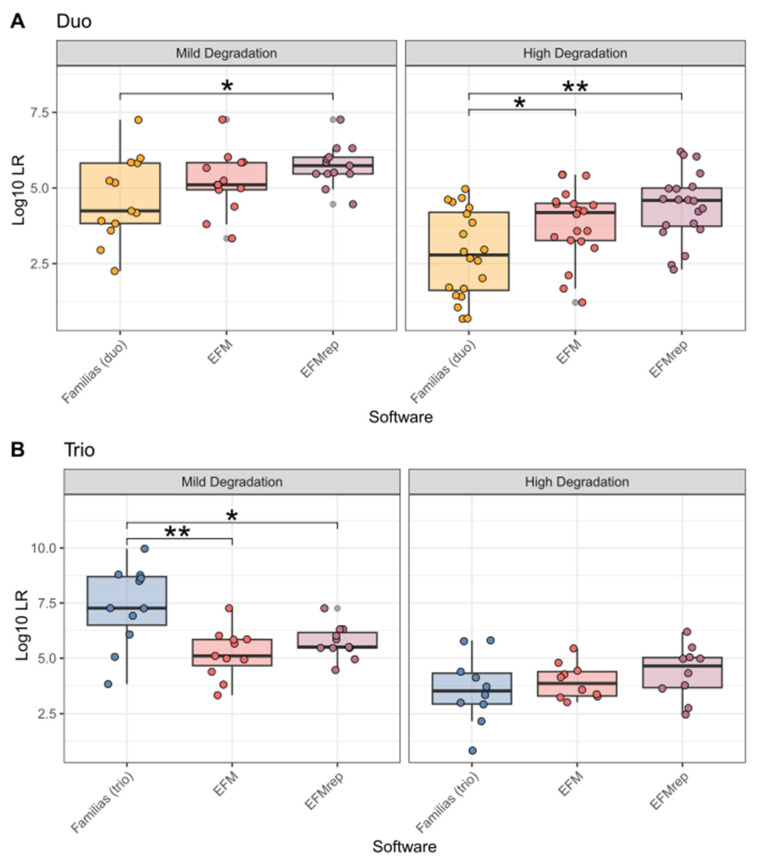
Boxplot showing LR values calculated by each software in duo (**A**) and trio (**B**) cases, divided by the degree of degradation of LT-DNA samples. Each dot represents a sample. “*”: *p* < 0.05, “**”: *p* < 0.01.

Comparisons of LR calculation results with standard kinship software and PGS are summarised in Figure 1.

It was found that, in duo cases, the LR values generated by EFMrep were significantly higher than those produced by Familias. This finding was consistent for both MD (*p* = 0.0002) and HD (*p* = 8.2 × 10^−5^) samples. Furthermore, for HD samples, the LR values generated by EFM were significantly higher than those of Familias (*p* = 0.0027), while no significant differences were detected between the two software programs for MD samples. In both cases, the LR values were significantly higher for EFMrep compared to EFM (MD *p* = 0.0012, HD *p* = 0.0064).

When maternal DNA information was available for MD samples (trios), the LR values produced by Familias were significantly higher than those obtained with EFM (*p* = 0.0049) and EFMrep (*p* = 0.0140). Within the MD samples, EFMrep also yielded significantly higher LR values than EFM (*p* = 0.0049). In contrast, no statistically significant differences among the three software were detected in the HD trio samples.

**Figure 2:**
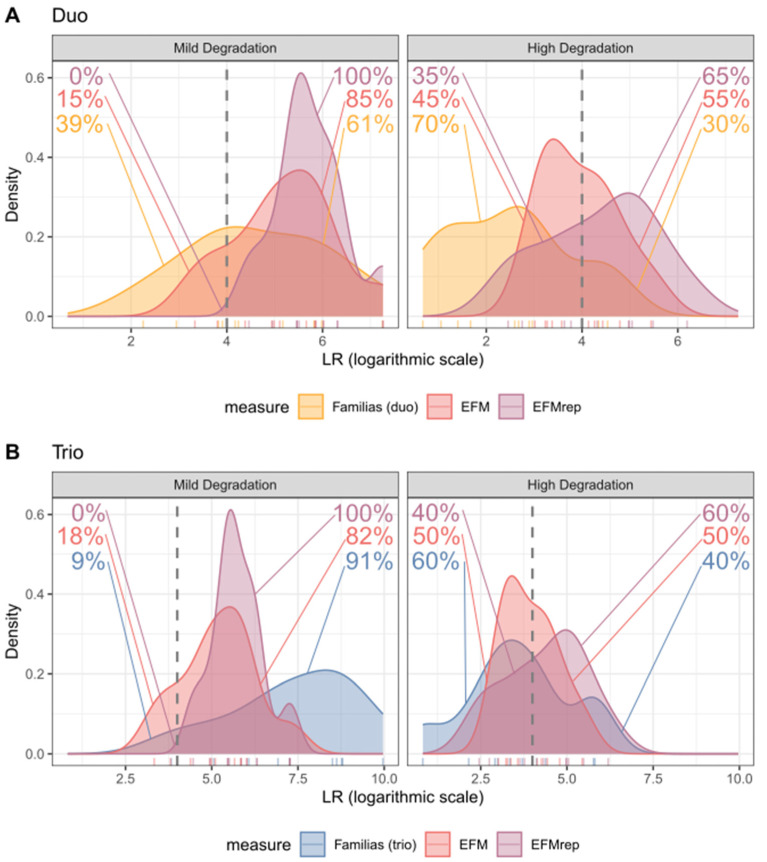
Density plot showing the LR values generated by each software in duo (**A**) and trio (**B**) cases, subdivided by the degree of degradation of the LT-DNA samples. The dashed line corresponds to LR = 10,000.

Figure 2 reports the distribution of LR values obtained with the three software in the different pedigree and degradation scenarios, highlighting the proportion of results below and above a set threshold of LR ≥ 10,000.

Given the significant differences in the paired LR values between the three software in the MD-duo samples, EFMrep consistently yielded LR values exceeding 10,000 (100%), while EFM showed a lower percentage (84.6%), which was even lower for Familias (61%), as shown in Figure 2A. Similarly, for HD-duos, EFMrep showed a larger proportion of cases exceeding the LR ≥ 10,000 threshold (65%) compared to EFM (55%) and Familias (30%). In MD-trio cases, although Familias produces on average higher LR values compared to PGS, it could be seen that, in 9% of the cases, Familias’ LR values were below 10,000, whereas values obtained with EFMrep always passed the set threshold. Finally, for HD-trios, as shown in Figure 2B, where all three software did not show significant differences in terms of LR values, EFMrep showed a larger proportion of cases exceeding the LR ≥ 10,000 threshold (60%) compared to EFM (50%) and Familias (40%).

**Figure 3:**
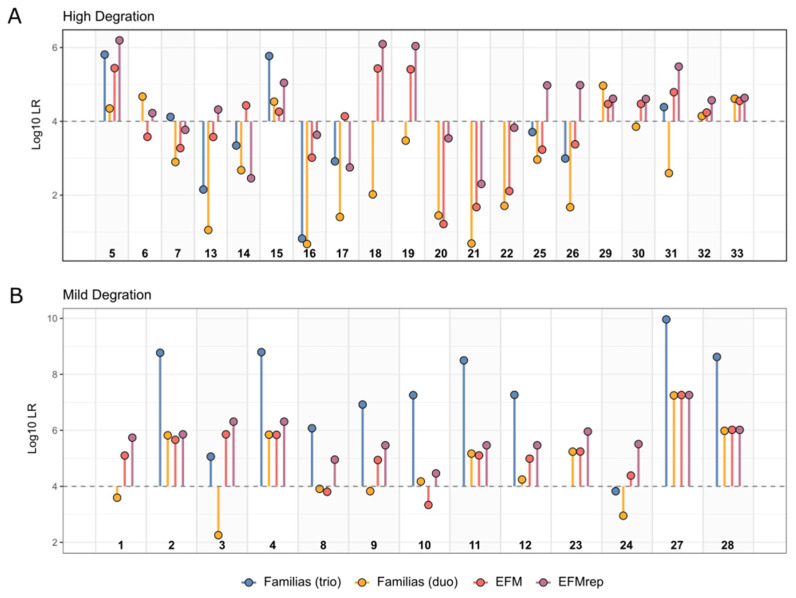
Lollipop plot of LRs computed with different software in HD (**A**) and MD samples (**B**). For the Familias software, LR values obtained taking into account maternal information when available (trio cases) are shown in blue. The dashed horizontal line indicates the threshold value of LR = 10,000. Sample ID numbers are shown below and correspond to those listed in Supplementary Table S1.

LR values obtained in each LT-DNA paternity case are depicted in Figure 3. As summarised in Table 4, the maximum difference between the LR values produced for the same LT-DNA samples by the three different software expressed in orders of magnitude (ΔLR max) was 4. Table 4 also shows that, for all pedigree and degradation scenarios except for MD-trios, the software most likely to maximise LR values was EFMrep. On the other end, in the majority of MD-trios (82%), the software that generated the highest LRs was Familias. Overall, it could also be seen that in cases where the LR values obtained with Familias fell below the 10,000 threshold (n = 10, 30%), LR values above 10,000 could be obtained with PGS. In particular, LR values ≥10,000 were observed with EFM in 20% of the cases (n = 2), with EFMrep in 20% of the cases (n = 2) and with both software in the remaining 60% of the cases (n = 6).

## 4. Discussion

The LT-DNA paternity cases selected for the study had all produced LR values > 1, thus supporting the hypothesis of paternity rather than the alternative (unrelatedness) [36], when kinship software Familias and standard internal procedures, consisting of two replicate PCR experiments of the same STR kit, were applied. This approach relied on the combination of a well-established method to model for drop-out implemented in Familias [16,37] and the classic binary ‘biological’ solution strategy to LT-DNA samples, which deduces a consensus profile from replicate analyses based on all-or-none allele detection [1,33,38]. Reanalysis of STR data using PGS generated outputs that were always in agreement with Familias in producing LR values > 1. Application of probabilistic genotyping to paternity analysis thus met the fundamental prerequisite for the internal validation of new software, that is, the consistency of results with those obtained on representative in-house generated data using standard laboratory-defined interpretative procedures [39].

Further steps of the software implementation process include the determination of robustness and limits in order to establish the types of profiles that are suitable for handling by the laboratory. The first factor to consider is whether the pedigrees being tested are standard trios or motherless cases (duos). In duo cases, uncertainty regarding the child’s maternal obligate alleles produces a loss of information with dual effect [40]. A general reduction in average LR values is observed, potentially leading to false-negative results, i.e., true fathers who do not reach the set LR threshold for inclusion [41]. Among the tested software programs, only Familias can take maternal information into account and, consequently, the higher LR values observed for Familias compared to EFM and EFMrep were expected, at least for cases in which degradation was mild and the effect of stochastic events was only marginally relevant.

The second effect of missing maternal information is the increased risk of false inclusions due to a reduction in the number of expected allelic inconsistencies between the child and an unrelated individual [42]. Thanks to the evolution of STR kits, which presently include more than 15 loci, it is now possible to overcome this pitfall of duo paternity testing, provided that the analysis is conducted on high-quality samples [43,44]. However, in LT-DNA samples, the number of STRs that can be genotyped effectively is often <15 due to fragmentation of template DNA. The problem is particularly evident when an extremely conservative approach (2×) is adopted to establish consensus profiles in Familias, as in this study. Previously, it was shown that the 2× method only detects an average of 50% of DNA profile alleles, which corresponds to the expected dropout of one-quarter of tested loci in LT-DNA conditions [33]. We have observed that, by applying probabilistic genotyping methods, the number of informative loci was significantly higher compared to Familias and was positively correlated with LR values. On average, the maximum number of informative STR loci and, consequently, significantly higher LRs were obtained with EFMrep, which leverages the combination of the results of PCR replicates obtained with different STR kits. The ESI and ESX STR kits employed in this research for EFMrep calculations, while targeting the same panel of loci, adopt distinct combinations of PCR primers [25]. Their combined use is particularly advantageous for the analysis of degraded or challenging samples, as it maximises the recovery of allelic information across loci and enables the confirmation of the results obtained with the alternate system [45].

In trio cases with highly degraded DNA, full quantitative analysis implemented in PGS compensated for the impossibility to take maternal information into account, producing LR values which were equivalent to those achieved with Familias, according to paired comparisons. However, in 80% of the cases, it was either EFM or EFMrep to maximise LR values.

Overall, irrespective of degradation status and pedigree type, in cases where Familias did not reach the nominal LR threshold of 10,000 for paternity inclusion according to national guidelines, this was possible by applying PGS. This threshold was set by policy based on high-quality DNA samples and standard LR calculation methods, and it represents a trade-off between the risks of false-positive and false-negative paternity test results [31,32]. It was observed that PGS could produce LR values up to four orders of magnitude larger than those calculated using Familias. It must be noted that the LR value produced by PGS and used for comparisons within the present study is of the MLE type. The determination of a more conservative LR through a sensitivity test, using the distribution of LRs based upon the errors of the parameters that were estimated to produce the MLE [23], is not feasible in EFM for calculations implying relatedness, and it is not implemented in the currently available version of EFMrep. This could potentially lead to an overestimation of evidence in favour of paternity. On the other hand, a risk of factitious underestimation of LR values when using PGS in paternity testing is linked to possible father–child mutations. The PGS investigated in the present study do not incorporate mutation rates, which range between 0.64% of STR SE33 and 0.01% of STR TH01 [34]. Consequently, any mismatch between the STR profiles of the LT-DNA sample and of the alleged father–child can only be interpreted as a PCR artefact. Consider, for example, a paternal LT-DNA sample displaying a heterozygous genotype that does not share any alleles with the alleged child. If paternity is hypothesised, with PGS used in the present study, the only possible explanation would be the drop-out of the shared allele together with the drop-in of a random allele. However, if the peak height of the allele assumed to have dropped in is significantly higher than the AT, the probability of this occurring, as calculated using the exponential drop-in function in EFM and EFMrep, is expected to be extremely low. This would result in an LR value that strongly rejects the proposition of paternity for the affected locus. If the paternal LT-DNA sample is homozygous at a given STR locus with no shared allele with the child’s genotype, one possible explanation under the proposition of paternity is that there was a drop-out of the locus accompanied by a drop-in of the observed allele. The effect on locus-specific LR results in this case will be the same as in the previous example. Another possible explanation would be drop-out in the paternal heterozygous genotype affecting the allele that is shared with the child. In this case, too, if the peak height of the paternal allele observed in the LT-DNA sample is well above the AT, the probability of drop-out of companion allele according to the continuous peak height model implemented in EFM and EFMrep is expected to be exceedingly low and the locus-specific LR will strongly support non-paternity. In any case, the model validation function implemented in EFM and EFMrep can be conveniently used to detect such events. It produces probability–probability plots that compare two empirical cumulative distributions of the observed peak heights with the theoretical underlying model. For reasonable peak height models, the probability values of STR loci will be arranged along a 45◦ straight line relationship, whereas single-STR loci affected by mutation will stand out as outliers.

We are aware that the PGS considered here (EFM and EFMrep) were not designed for the purpose of paternity testing. However, it has been suggested that statistical redundancy, i.e., applying a multi-software approach to challenging forensic cases, can be beneficial for the interpretation of the data and provide an additional diagnostic check [46,47]. The obtained results shed light on the potentialities and constraints of the statistical models underlying standard kinship software and PGS and contribute to the needed interpretational background required for the potential future use of PGS as a supplementary tool in paternity testing of LT-DNA samples [48].

Finally, it is important to note that the present study has limitations. Firstly, EFM and EFMrep are not the only PGS programs that can take the relatedness of DNA profile donors into account, so comparisons could be extended to other software with similar properties described in the literature [49,50]. Secondly, the analysis was conducted under simplified experimental conditions, i.e., only two PCR replicates were obtained using two STR kits with a limited number of loci (16). Given that a concrete balance must be reached in forensic investigations between test informativity and laboratory costs [51], it is known that by increasing the number of PCR replicates, it is possible to boost robustness and reliability [33,52]. While the use of multiplex PCR systems with additional STRs does not seem to provide a substantial gain in the number of detected alleles and informative loci in LT-DNA samples [53], the effect of combined results from different expanded STR kits has not been investigated so far. So, it could be interesting in the future to extend this study to include more PCR replicates and, for EFMrep, combinations of STR kits. The investigation of the performance of standard paternity software and PGS in more complex kinship scenarios, different from paternity, could be another intriguing development.

## 5. Conclusions

This preliminary study represents a first step for the internal validation of PGS in paternity investigations of LT-DNA samples and for the definition of an operational flow chart to identify the combination of calculation software most suitable for the starting analytical conditions in terms of the type of pedigree and the level of DNA degradation.

## Figures and Tables

**Table 1:** Characteristics of the software used in this study for the analysis of LT-DNA samples in paternity cases.

	Familias	EFM	EFMrep
Drop-out	Yes	Yes	Yes
Drop-in	No	Yes	Yes
Stutter	No	Yes	Yes
Mutation	Yes	No	No
Maternal information	Yes	No	No
Peak height	No	Yes	Yes
PCR replicas	No (consensus)	Yes (single STR kit)	Yes (multiple STR kits)

**Table 2:** Method used to create consensus STR profiles and to assign dropout probabilities.

Peaks in Replicate 1	Peaks in Replicate 2	Consensus Genotype	Drop-Out Probability
A	None or B	None	Locus not considered in calculations
A	A	A–A	Calculated according to logistic regression, using the highest A allele peak among the two replicates
A–B	A	A–A	Calculated according to the logistic regression of A allele peak of replicate 2
A–B	A–C	A–A	Calculated according to logistic regression using the lowest A allele peak among the two replicates
A–B	A–B	A–B	No drop-out

**Table 3:** Mean number of informative STR loci (±SD) for the three software in MD and HD LT-DNA cases.

	Familias	EFM	EFMrep
MD (n = 13)	15 ± 1.6	15.6 ± 1.1	15.9 ± 0.3
HD (n = 20)	10.4 ± 4.3	14 ± 2.2	15 ± 1.2

**Table 4:** ΔLR max values observed with the three software in sample categories subdivided by LT-DNA degradation level and pedigree type, shown on the left. Columns on the right report the percentage of cases in which, within a specific scenario, the indicated software produced the highest LR value.

	ΔLR Max	Familias	EFM	EFMrep
MD-duo (n = 13)	4	0%	15%	85%
HD-duo (n = 20)	4	10%	10%	80%
MD-trio (n = 11)	4	82%	0%	18%
HD-trio (n = 10)	3	20%	20%	60%

## Data Availability

General characteristics and LR values of anonymized LT-DNA cases used in this study can be found in the Supplementary Materials.

## Supplementary Materials

The following supporting information can be downloaded at https://www.mdpi.com/article/10.3390/genes17020187/s1. Figure S1: Probability of drop-out as a function of peak height expressed in RFU for the PowerPlex ESI 17 Fast (A) and ESX 17 Fast (B) kit. Figure S2: Probability of drop-in as a function of peak height expressed in RFU for the PowerPlex ESI 17 Fast (A) and ESX 17 Fast (B) kit. Table S1: General characteristics of tested LT-samples: sample number, sample source, degradation status (MD: mildly degraded; HD: highly degraded), type of pedigree (duo, trio), number of informative STRs and LR values obtained with the software Familias, EuroForMix, and EFMrep. For trio cases, Familias LRs calculated both considering and disregarding maternal information are given.

## Abbreviations

The following abbreviations are used in this manuscript:

5ST	5% Stochastic Threshold
AT	Analytical threshold
EFM	EuroForMix
EDNAP	European DNA Profiling Group
ENFSI	European Network of Forensic Science Institutes
ESI	PowerPlex^®^ ESI 17 Fast kit
ESX	PowerPlex^®^ ESX 17 Fast kit
ESS	European Standard Set
FST	Fixation Index (population genetics correction)
FFPE	Formalin-Fixed, Paraffin-Embedded
HD	Highly Degraded
Hd	Prosecution Hypothesis (paternity/relatedness)
Hp	Defence Hypothesis (alternative/unrelated)
ISFG	International Society for Forensic Genetics
LR	Likelihood Ratio
LT-DNA	Low-Template DNA
MD	Mildly Degraded
MLE	Maximum Likelihood Estimation
PGS	Probabilistic Genotyping Software
POI	Person of Interest
RFU	Relative Fluorescence Units
STR	Short Tandem Repeat
