# Supplementary material for: Application of Probabilistic Genotyping Software to Paternity Cases Involving Low-Template DNA
Source: Genes (Basel). 2026 Feb 1;17(2):187. doi: 10.3390/genes17020187 (PMC12940192; doi:10.3390/genes17020187)
Supplement: Supplementary file 1 [file genes-17-00187-s001.zip › Captions.pdf]

Figure S1: Probability of drop-out as a function of peak height expressed in RFU for the PowerPlex ESI 17 Fast (A) and ESX 17 Fast (B) kit.

Figure S2: Probability of drop-in as a function of peak height expressed in RFU for the PowerPlex ESI 17 Fast (A) and ESX 17 Fast (B) kit.

Table S1: General characteristics of tested LT-samples: sample number, sample source, degradation status (MD: mildly degraded; HD: highly degraded), type of pedigree (duo, trio), number of informative STRs and LR values obtained with the software Familias, EuroForMix, and EFMrep. For trio cases, Familias LRs calculated both considering and disregarding maternal information are given.
