# Supplementary figures and images for: Application of Probabilistic Genotyping Software to Paternity Cases Involving Low-Template DNA
Source: Genes (Basel). 2026 Feb 1;17(2):187. doi: 10.3390/genes17020187 (PMC12940192; doi:10.3390/genes17020187)

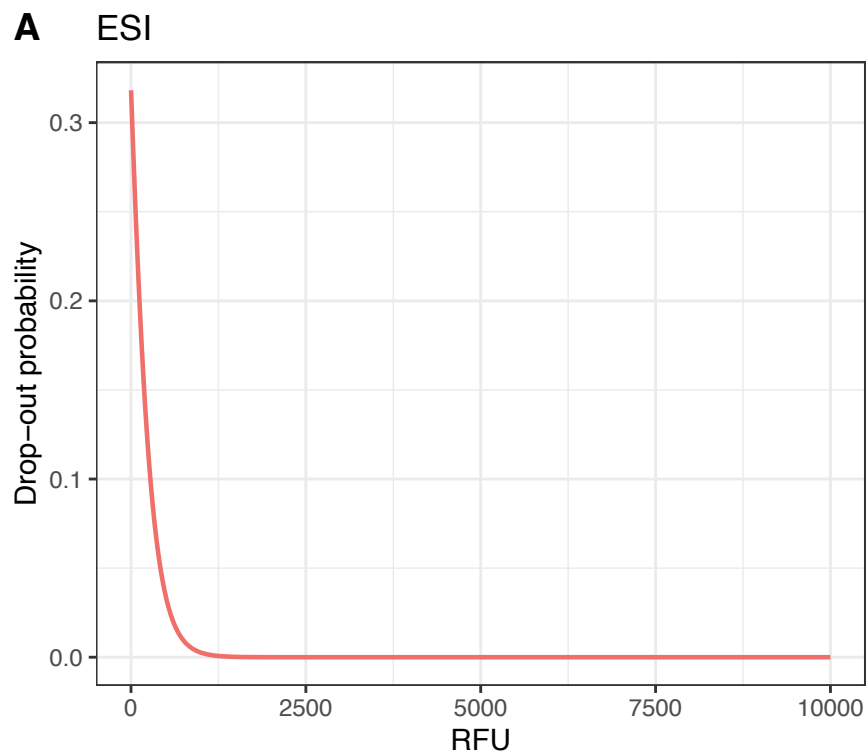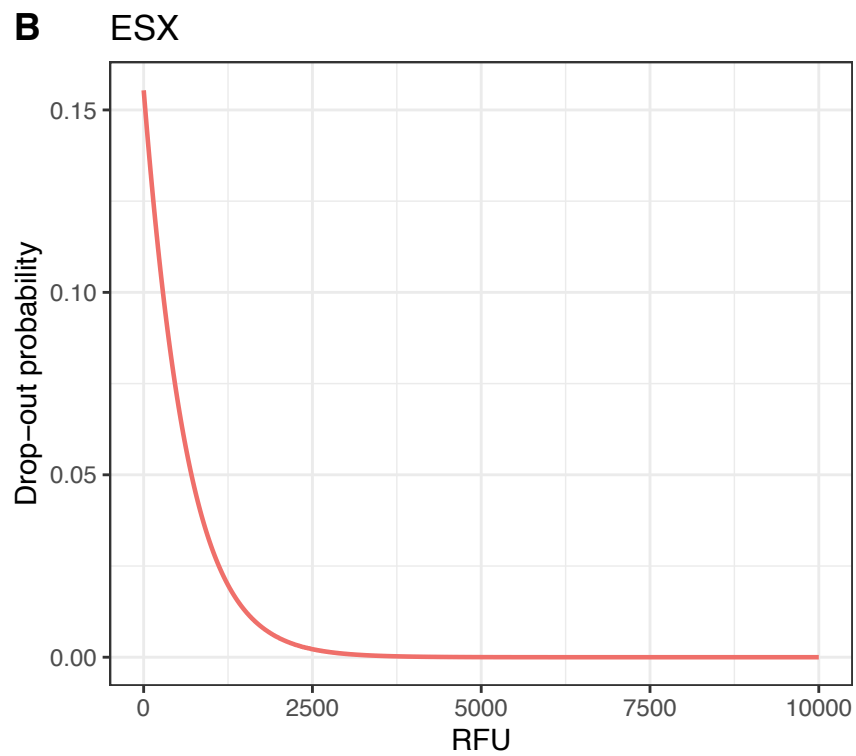

Supplement: Supplementary file 1 [file genes-17-00187-s001.zip › Supplementary_Figure_S1.pdf]

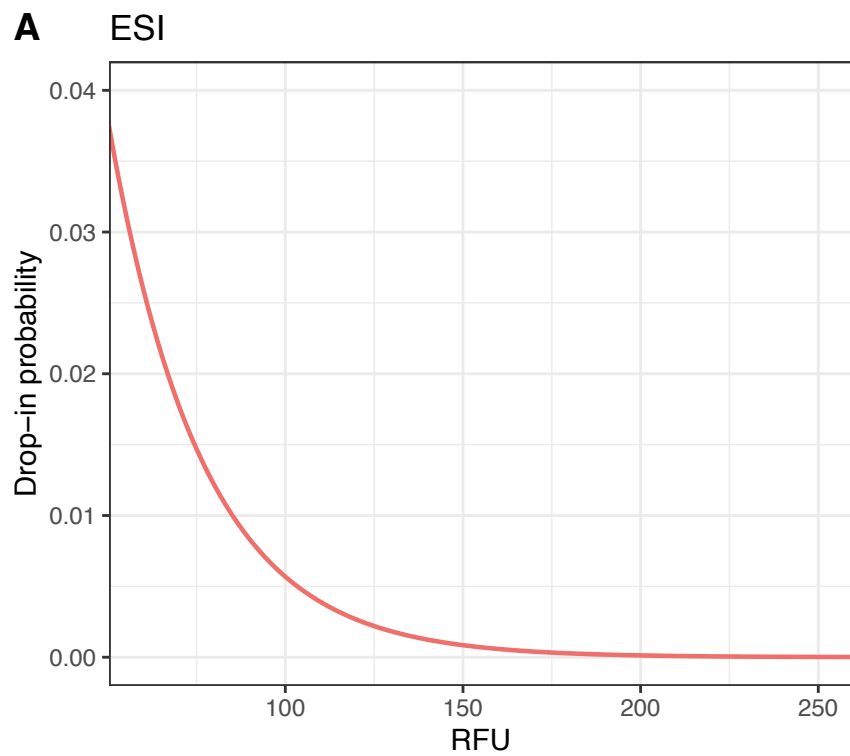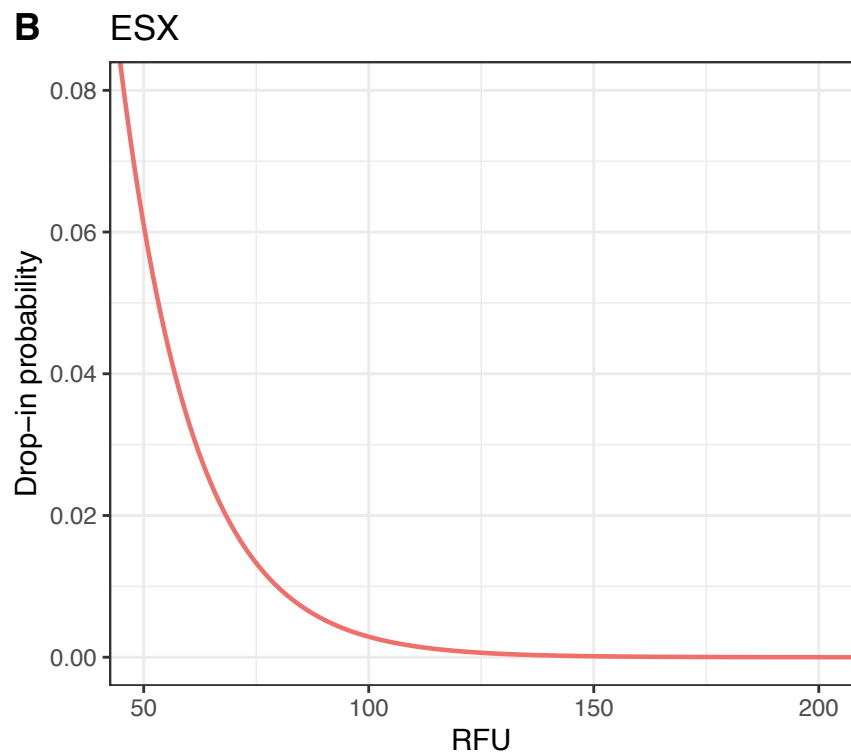

Supplement: Supplementary file 1 [file genes-17-00187-s001.zip › Supplementary_Figure_S2.pdf]
